# Vendor of choice and the effectiveness of policies to promote health information exchange

**DOI:** 10.1186/s12913-018-3230-7

**Published:** 2018-06-04

**Authors:** Anabel F. Castillo, Marvin Sirbu, Alexander L. Davis

**Affiliations:** Department of Engineering and Public Policy, Carnegie Mellon University, 5000 Forbes Avenue, Pittsburgh, PA 15213 USA

**Keywords:** Electronic Health Records, Interoperability, EHR vendors, Meaningful use

## Abstract

**Background:**

As more hospitals adopt Electronic Health Records (EHR), focus has shifted to how these records can be used to improve patient care. One barrier to this improvement is limited information exchange between providers. In this work we examine the role of EHR vendors, hypothesizing that vendors strategically control the exchange of clinical care summaries. Their strategy may involve the creation of networks that easily exchange information between providers with the same vendor but frustrate exchange between providers with different vendors, even as both Federal and State policies attempt to incentivize exchange through a common format.

**Methods:**

Using data from the 2013 American Hospital Association’s Information Technology Supplement, we examine the relationship between a hospital’s decision to share clinical care summaries outside of their network and EHR vendor market share, measured by the percentage of hospitals that have the same vendor in a Hospital Referral Region.

**Results:**

Our findings show that the likelihood of a hospital exchanging clinical summaries with hospitals outside its health system increases as the percentage of hospitals with the same EHR vendor in the region increases. The estimated odds of a hospital sharing clinical care summaries outside their system is 5.4 (95% CI, 3.29–8.80) times greater if all hospitals in the Hospital Referral Region use the same EHR Vendor than the corresponding odds for a hospital in an area with no hospitals using the same EHR Vendor. When reviewing the relationship of vendor market concentration at the state level we find a positive significant relationship with the percentage of hospitals that share clinical care summaries within a state. We find no significant impact from state policies designed to incentivize information exchange through the State Health Information Exchange Cooperative Program.

**Conclusion:**

There are benefits to exchanging using proprietary methods that are strengthened when the vendors are more concentrated. In order to avoid closed networks that foreclose some hospitals, it is important that future regulation attempt to be more inclusive of hospitals that do not use large vendors and are therefore unable to use proprietary methods for exchange.

**Electronic supplementary material:**

The online version of this article (10.1186/s12913-018-3230-7) contains supplementary material, which is available to authorized users.

## Background

Exchange of health information through interoperable systems is an essential goal as providers transition from hard to digital copies of medical records [1]. Interoperable systems ensure that electronic health information can be used and exchanged without any special effort from the sender or receiver through the use of a common language [2]. Without interoperable systems, the full potential benefits of adopting Electronic Health Records cannot be achieved [3].

The Health Information Technology for Economic and Clinical Health (HITECH) Act enacted in 2009 [4], and the associated State Health Information Exchange Cooperative Program [5], provide monetary incentives to eligible providers and hospitals to support the adoption of EHRs and health information exchange. To receive these incentives, hospitals and physicians must meet usability criteria also known as meaningful use (MU) objectives (core and menu) that ensure EHRs are used to support health policy priorities [6]. EHR products that are purchased through the Meaningful Use incentives are certified by the Department of Health and Human Services [7]. While certification criteria changed the supply side of the EHR market, the stated incentives allowed for a greater demand for EHR certified products [8].

A core objective of Meaningful Use’s second stage is the exchange of clinical summaries when patients transition between providers. Until the year 2014, certification requirements stated that both the Continuity of Care Record (CCR) and the Continuity of Care Document (CCD) standardized formats could be used for said exchange [9]. Current regulation, published in 2015, requires that vendors demonstrate that they are able to use the second version of the Consolidated Clinical Document Architecture (C-CDA 2.1) markup standard. Aside from the format standard, MU incentives foster an open approach to health information exchange, allowing for direct exchange among EHR vendors as well as enterprise and community solutions [10].

Despite these efforts to promote interoperability and meaningful use, information exchange has remained below expectations set by HITECH [1, 11, 12]. Qualitative and quantitative studies identify several operational and economic barriers to health information exchange. Qualitative studies have found that operational barriers include the use of information as a competitive advantage, lack of cost-efficiency, limited incentives for staff and diminished trust of other providers [13–15]. Quantitative studies have shown that certain hospital characteristics are related to the probability that hospitals exchange health information with other hospitals. For example, Adler-Milstein and Jha [16] found that hospitals with a larger market share within a region were more likely to participate in information exchange but that hospitals in competitive markets were less likely to share information. Furthermore, Miller and Tucker [17] found that hospitals that are part of larger health systems are less likely to exchange information with hospitals outside their system. In sum, several studies find that information exchange is a function of a hospital’s strategic reasons for sharing [16–20].

Although most research has looked at the characteristics of hospitals associated with information exchange, recently more focus has been directed toward vendors and how they may also use information exchange strategically [21]. While EHR products must be capable of transforming from proprietary architectures to the semantics and structure used in CCR or CCD formats at least once during the certification process, there are still reports of both cost and technical barriers for the process of exchanging clinical care summaries [22, 23]. Hence, EHR vendors could knowingly and unjustifiable interfere with health information exchange by engaging in what is known as information blocking [21].

We hypothesize that vendors can leverage proprietary software to make it easier for hospitals to share clinical care summaries with other hospitals that have the same software while making it challenging to share with hospitals that use a different EHR vendor. This imposes costs on hospitals that need to share information and creates an incentive for them to adopt the dominant vendor. Specifically, research has shown that hospitals that use dominant vendors likely face fewer technical obstacles and engage in a higher number of HIE activities [24].

Empirical work in other domains supports this. For example, Shapiro and Varian find that there are network externality benefits to being connected to a larger communication network [25]. The value of connecting to a network depends on the number of others that are already connected, which means that from the perspective of a user, being connected to a bigger network is better [25]. When an EHR vendor has a large closed network, in this case a large number of hospitals that use its product, a user will have an easier time sharing information. This creates a competitive advantage for the EHR vendor that provides the closed network.

Even if policy incentivizes the exchange of clinical care summaries, there is significant variation in the use of HIE across EHR vendors. Some vendors have been at the forefront by facilitating exchange through private proprietary networks. The most prominent of these networks is Care Everywhere, a system incorporated into EPIC EHR products since 2005 [26]. Although Care Everywhere is meant to be able to connect to EHR systems from other vendors, it is most successfully used to connect with other EPIC users [27]. Additionally, connection even within the Care Everywhere network may require additional customization [28].

The present study aims to identify the effect of vendor choice and vendor network size on whether a hospital reports participating in the exchange of clinical care summaries. As a measure of the size of an EHR vendor’s network we will use EHR vendor market share and market concentration. We hypothesize that *the probability of a hospital engaging in the exchange of clinical care summaries with another hospital outside its health systems is associated with the market share of the EHR vendor in the immediate region where exchanges are more likely to occur.*

To determine this association, we use a logistic regression model at the hospital level using the response from each provider about whether they exchange clinical care summaries outside of their system as a dependent variable. EHR vendor market share is measured by the percentage of hospitals that have the same vendor in the hospital referral region (HRR) where a hospital operates. These regions, or markets for tertiary medical care, consolidate zip codes where the majority of patients are referred to a specific hospital for cardiovascular surgery and neurosurgery [29]. We expect that in HRRs where intra-vendor sharing occurs the probability of a hospital engaging in information exchange increases as the market share of this hospital’s EHR vendor increases. This effect is due to the increase of the number of opportunities to engage in intra-vendor exchange. We also test for differences that might be unique to large EHR vendors that have established proprietary information networks, such as EPIC, by testing the different interactions in logistic regressions for the three largest EHR vendors.

A second analysis is done at the state level. The aim of this second model is to further analyze the propensity to share in the context of state level policies that incentivize health information exchange. Our hypothesis is that a higher EHR vendor market concentration, measured by the Herfindahl-Hirschman Index (HHI), is associated with a positive change in the percentage of hospitals that participate in information exchange within each state. We also expect to find differences in the propensity of this exchange depending on the strategies adopted by each state to incentivize HIE.

## Methods

### Data

We use data from the 2013 American Hospital Association (AHA) Annual Survey Information Technology Supplement. The survey was distributed between November 2013 and February 2014 to the Chief Executive Officers of U.S. Hospitals, who in turn may delegate the responsibility of completion to the institution’s qualified IT personnel. The survey had a response rate for non-federal acute care hospitals of 61% (2737/4451 hospitals). For our first model we drop 311 hospitals that are not able to generate summary of care records for transitions of care. We also remove the hospitals for which we have no information relevant to our main variables of analysis (electronically exchange clinical care summaries, EHR vendor, use of common format and ability to exchange with other EHR vendors). Finally, we drop regions with less than three hospitals and are left with a sample of 1871 acute care hospitals. Detailed characteristics of our final sample are included in the Additional Files used in the logistic regression. At the state level we aggregate the data from this survey to create indicators for the percentage of hospitals that participate in HIE exchange and the prevalence of EHR vendors in each state.

Additionally, we use data from the Healthcare Information and Management Systems Society (HIMSS) Analytics Database for the year 2012, which compiles data on the Information Technology capabilities for 5467 hospitals. From this database we extracted each hospital’s affiliation to an Integrated Delivery System (IDS). An IDS is a healthcare organization that owns at least two medical/surgical hospitals. In this analysis we will refer to an IDS as a health system. We also use reports from the Centers for Medicare and Medicaid Services (CMS) that detail the EHR Products used for meaningful use attestation by eligible hospitals. Finally we use reports from the Office of the National Coordinator for Health Information Technology (ONC) on the status of the State HIE Cooperative Agreement Programs in 2013.

### Measures

#### Dependent variables

##### Information Exchange (IE) and percent of hospitals that Exchange Information (%IE)

We use information exchange as our dependent variable, operationalized as the yes/no answers found in the AHA IT supplement database to questions about whether each hospital electronically exchanges/shares patient information such as laboratory results, medication history, radiology reports, and clinical care summaries with providers outside their health system. We use the exchange of clinical summaries during transitions of care, which is the requirement for Stage 2 meaningful use compliance, coded as one or zero for yes and no, respectively. According to the ONC, a clinical care summary includes basic clinical information regarding the care provided, such as medications, upcoming appointments, or other instructions. It is shared with patients and clinicians in order to increase awareness of what occurred during office visits and can be used to assist care coordination. This variable was used to determine a hospital’s indication of health information exchange (***IE***) and was also aggregated to determine the percentage of hospitals that answered positively to sharing within a state (***%IE***), using as a denominator the number of hospitals on the final sample (a total of 2296 hospitals).

##### Vendor Market Share (VMS)

To operationalize vendor market share, we used data from the AHA IT supplement database that requested the name of the hospital’s primary outpatient EHR/EMR tool. This data was checked and complemented with data from the CMS Meaningful Use Attestation database, which has information on the outpatient EHR product used by eligible hospitals that participate in the MU program. The indicator for EHR vendor market share (***VMS***) for each hospital was calculated by determining the percentage of hospitals within a Hospital Referral Region that use the same EHR vendor as the subject hospital.

##### State EHR Vendor HHI (VendorHHI)

To determine the EHR vendor market concentration in a state we use the Herfindahl-Hirschman Index (HHI), the standard measure used by the U.S. Department of Justice to determine market concentration [30]. This indicator measures market concentration using the relative size of the market share and distribution of the firms in a market [30]. For our analysis we define market share as the number of final users (patients) that will use a specific EHR Vendor. As a proxy for the number of patients we use the number of beds in each hospital, giving more weight to larger hospitals. We then calculate the HHI index by squaring the percentage share of beds for each EHR Vendor at the state level (***VendorHHI***).

#### Hospital-level variables

Other potential explanatory variables are extracted from the AHA IT Supplement database. We use an indicator for a hospital’s capability to send clinical summary of care records in one of three formats (***CL***): Continuous Care Record (CCR), Clinical Document Architecture (CDA) or Continuous Care Documentation (CCD). Also included is a variable that asks if the hospital’s EHR allows sending clinical care summaries to unaffiliated hospitals using a different EHR vendor (***CS***).

Other hospital descriptive indicators, which have been found significant in the literature, such as hospital size (***Size***) [17], ownership (***Ownership***), rural or urban location (***Rural***) and hospital HHI [16], are also included. Hospital HHI (***HospitalHHI***) at the regional level is calculated by weighting hospital market participation in an HRR with hospital size, using total beds as a proxy. To determine health system affiliation, we used the IDS indicator from the HIMSS analytics database and included a dummy variable that indicates if there is more than one hospital from the same health system in the HRR (***System Hospital***). The aim of this last indicator is to account for different sharing policies between hospitals that are part of the same system and are in the same region. Finally, we used dummy variables for the largest three EHR Vendors: Epic, Meditech and Cerner. We test the efficacy of state programs to encourage HIE by adding dummy variables indicating the availability (***RHIO***) and use (***RHIO***_***PART***_) of Regional Health Information Organizations (RHIO), organizations that bring together health care stakeholders within a defined geographic area and govern health information exchange among them [31].

#### State-level variables

Information on the models used by states for information exchange was extracted from the ONC progress report on the State HIE Cooperative Agreement Program [32]. We coded variables on the availability of Direct and Query exchange if the state reported that each type of exchange was “broadly available”. Broadly available types of exchange include Directed Exchange (point-to-point secure communication) and Query-based Exchange (pull transactions through a request) [33]. We also coded variables for the strategic approaches each state used to encourage information exchange, including four categories Elevator (rapid facilitator of Directed Exchange), Capacity Builder (assists regional exchanges through financial and technical support), Orchestrator (state level network to connect regional exchanges) and Public Utility (provides HIE services directly) [34].

Using the IDS indicator from the HIMSS analytics database we calculated an HHI index for Health Systems in a State (***SystemHHI***) also weighted by hospital beds. We also included a variable for the number of beds in a State (***HospitalsState***).

#### Analyses

To determine the relationship between the probability of a hospital engaging in information exchange and EHR Vendor market share we used a logistic regression model. The basic bivariate model between the dependent variable Information Exchange (***IE***) and our variable of interest Vendor Market Share (***VMS***) Concentration is represented by eq. 1.

1 $$ logit\left( IE| VMS\right)=\log \left(\frac{P\left( IE| VMS\right)}{1-P\left( IE| VMS\right)}\right)\kern1.5em ={\beta}_0+{\beta}_1\ast VMS $$

$$ p\left( IE| VMS\right)=\kern0.5em logistic\ equation $$

We then added other explanatory variables found in the literature to reduce possible omitted variable bias (eq. 2).

2 $$ \log \left(\frac{\widehat{p}(x)}{1-\widehat{p}(x)}\right)=\widehat{\eta}(IE) $$

$$ \widehat{\eta}(IE)={\widehat{\beta}}_0+{\widehat{\beta}}_1\ast VMS\kern0.5em +{\widehat{\beta}}_2\ast CL\kern0.5em +{\widehat{\beta}}_3 CS+{\widehat{\beta}}_4\ast RHIO+{\widehat{\beta}}_5\ast {RHIO}_{PART}+{\widehat{\beta}}_6\ast Ownership\kern0.5em +{\widehat{\beta}}_7\ast Rural\kern0.5em +{\widehat{\beta}}_8\ast Size+{\widehat{\beta}}_9\ast System\ Hospital\kern0.5em +{\widehat{\beta}}_{10}\ast HospitalHHI+\varepsilon $$

We also include state fixed effects to control for local characteristics that might impact information exchange and analyzed the characteristics for the largest market players by including dummy variables.

For our second analysis we looked for an association between vendor concentration and the percentage of hospitals that exchange clinical care summaries within a state. To test this association, we used a multivariate linear regression model represented by eq. 3.

3 $$ \% IE={\widehat{\beta}}_0+{\widehat{\beta}}_1\  VendorHHI+{\widehat{\beta}}_2\  SystemHHI+{\widehat{\beta}}_3\  HospitalsState+\varepsilon $$

To this model we added dummy variable indicators for state level policies to incentivize health information exchange.

## Results

### Vendor market share and hospital information exchange

#### Logistic hospital level regression

**Fig. 1:**
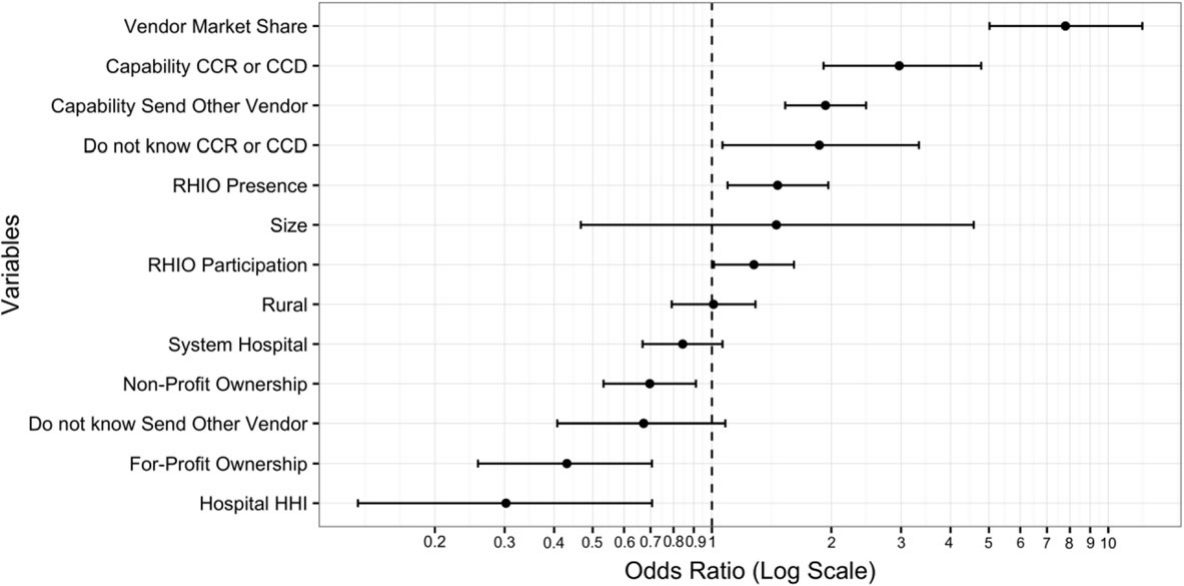
Odds Ratio for Independent Variables Predicting Probability that a Hospital “Shares Clinical Care Summary Outside their Health System” with error bars for a 95% interval

We find that for our 2013 dataset there was a positive relationship between the likelihood of sharing and a hospital’s EHR vendor market share within a HRR. A graphic representation of the logistic regression results can be seen in Fig. 1, which presents the odds ratio results of the logistic regression with error bars representing a 95% confidence interval. Additionally, in an effort to control for the different state level characteristics that might influence the likelihood of hospital sharing we used a state fixed effects (Table 1) again finding the same positive relationship between VMS and information exchange. The location of the hospital was determined by the provided zip code address as HRRs are regions of service provision and therefore are not always within state boundaries.

**Table 1:** Adjusted odds ratio for hospitals with dependent variable “Shares Clinical Care Summary Outside their Health System” with state fixed effects

	Hospital shares clinical summary	
Variables	Odds ratio	95% confidence interval
Vendor market share	5.37^***^	(3.29, 8.80)
CCR or CCD (YES)	3.19^***^	(2.00, 5.27)
Allow other EHR Vendor (YES)	1.90^***^	(1.47, 2.45)
RHIO	1.23	(0.90, 1.67)
RHIO participation	1.56^***^	(1.19, 2.04)
Non-Profit ownership	0.69^**^	(0.51, 0.94)
For-Profit ownership	0.57^**^	(0.33, 0.96)
Rural	0.84	(0.63, 1.11)
Number of beds	3.28^*^	(0.94, 11.6)
System hospital	0.87	(0.68, 1.11)
Hospital HHI	0.62	(0.23, 1.67)

Note:^*^p < 0.1; ^**^p < 0.05; ^***^p < 0.01

When we control for State fixed effects, the estimated odds of a hospital sharing clinical care summaries outside their system is 5.4 times greater if all hospitals in the HRR use the same EHR Vendor than the corresponding odds for a hospital in an area with no hospitals using the same EHR Vendor. We include dummy variables for the ability to send documents in CCR or CCD format, if an EHR system allows for sending summary of care records to another EHR vendor and the availability of RHIOs. These three variables are significant in increasing the likelihood of sharing clinical summaries; nevertheless, the effect of Vendor Concentration remains large in comparison.

The results remain stable as we include other control variables that have been found relevant in the literature such as ownership (non-profit versus for-profit), rural versus urban location, normalized hospital size, hospital market concentration, and system affiliation. Of these only rural status and system affiliation were not statistically significant.

For-profit hospitals are found to be less likely to share information, which is consistent with the results found by Adler-Milstein and Jha [16] who hypothesize that a hospital’s strategic decision not to participate in information exchange is an effort to minimize costs. We also find that the measure of hospital market concentration is negatively related to the probability of participating in the exchange of clinical care summaries, which suggests that hospitals in more concentrated markets are less likely to exchange information.

#### Differences between specific vendors

Three EHR vendors, Epic, Meditech and Cerner, together control 58% of the hospital market in our sample data. Of the pool of non-federal acute hospitals that responded to the survey question, 39% had shared clinical care summaries with outside hospitals. We find that hospitals that use Epic exchange clinical care summaries significantly more than the total average, while hospitals that use Meditech or Cerner do so significantly less (Table 2). The type of hospitals that chose a specific vendor also varies between the different EHR vendors (Table 3).

**Table 2:** Percent of hospitals that share clinical care summaries outside their health system for the seven largest vendors

Vendor	% share clinical care summary	*n*(*N*)	*p* value^*^ (two-tailed)
EPIC	73%	296(407)	*p* < 0.01
Meditech	27%	97(360)	*p* < 0.01
Cerner Corporation	32%	109(338)	*p* < 0.01
McKesson	30%	58(191)	*p* < 0.01
CPSI	30%	48(160)	*p* < 0.05
Siemens	40%	41(102)	
Allscripts	26%	19(74)	*p* < 0.05

*Using t-test for equality of means

*n* = Number of hospitals that share clinical care summaries with hospitals outside their system

*N* = Total number of hospitals that use each EHR Vendor included in the database and that responded to the variable of analysis

**Table 3:** Differences in hospital characteristics of hospitals for Epic, Meditech and Cerner

		All EHR vendors	EPIC	Meditech	Cerner
		*N* = 1871	*N* = 407	*N* = 360	*N* = 338
Hospital size	Small (< 100 beds)	47%	33%^***^	44%	33%^***^
	Medium (100–399 beds)	40%	44%^*^	51%^***^	49%^***^
	Large (> = 400 beds)	13%	23%^***^	5%^***^	18%^***^
Ownership	Non-Profit	71%	88%^***^	69%	78%^***^
	For-Profit	8%	1%^***^	14%^***^	7%
	Public	21%	11%^***^	17%^**^	15%^***^

Note: Using t-test for equality of means the significance levels for a two-tailed tests are *p < 0.1; **p < 0.05; ***p < 0.01

Our results from the logistic regression show that hospital size is positively related to information exchange while being a For-Profit hospital is negatively related to this variable. In Table 3 we see that hospitals that use Epic as their EHR vendor are significantly larger and less likely to have a For-Profit ownership model. This is consistent with being more likely to share information. The opposite is true for hospitals that use Meditech, which are significantly less likely to be large and more likely to be For-Profit.

**Fig. 2:**
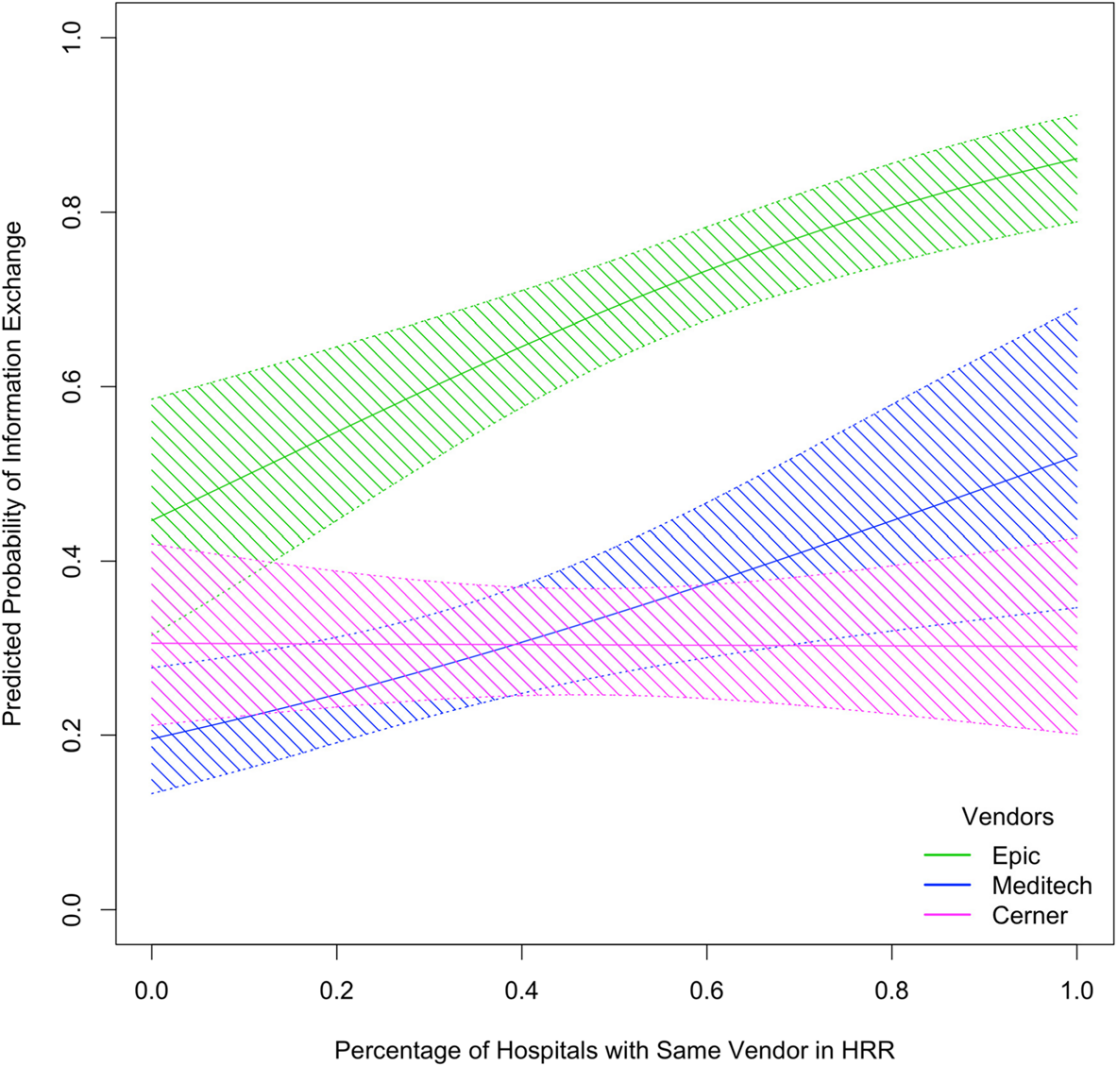
Predicted Probability that a Hospital “Shares Clinical Care Summary Outside their Health System” for Each of the Three Largest Vendors with 95% confidence interval

We expect that these three vendors have the potential of exploiting the network effects of market concentration because of their large number of users. We ran separate regressions to test the interactions between the main EHR vendors and the variable of interest. From Fig. 2 we find that there are important differences in the coefficient of the key independent variable Vendor Market Share for each of the different EHR vendors. Although hospitals using Epic start with a higher predicted probability of sharing, the increase of market share in the HRR has an important positive effect. A similarly positive effect is found for hospitals using Meditech as their EHR Vendor. However, for hospitals that use Cerner find there are negative effects of having other hospitals with the same vendor in the HRR. This suggests additional non-measured difficulties in information exchange for Cerner users.

### Vendor concentration at the state level

#### Percent sharing within state and EHR vendor concentration

**Fig. 3:**
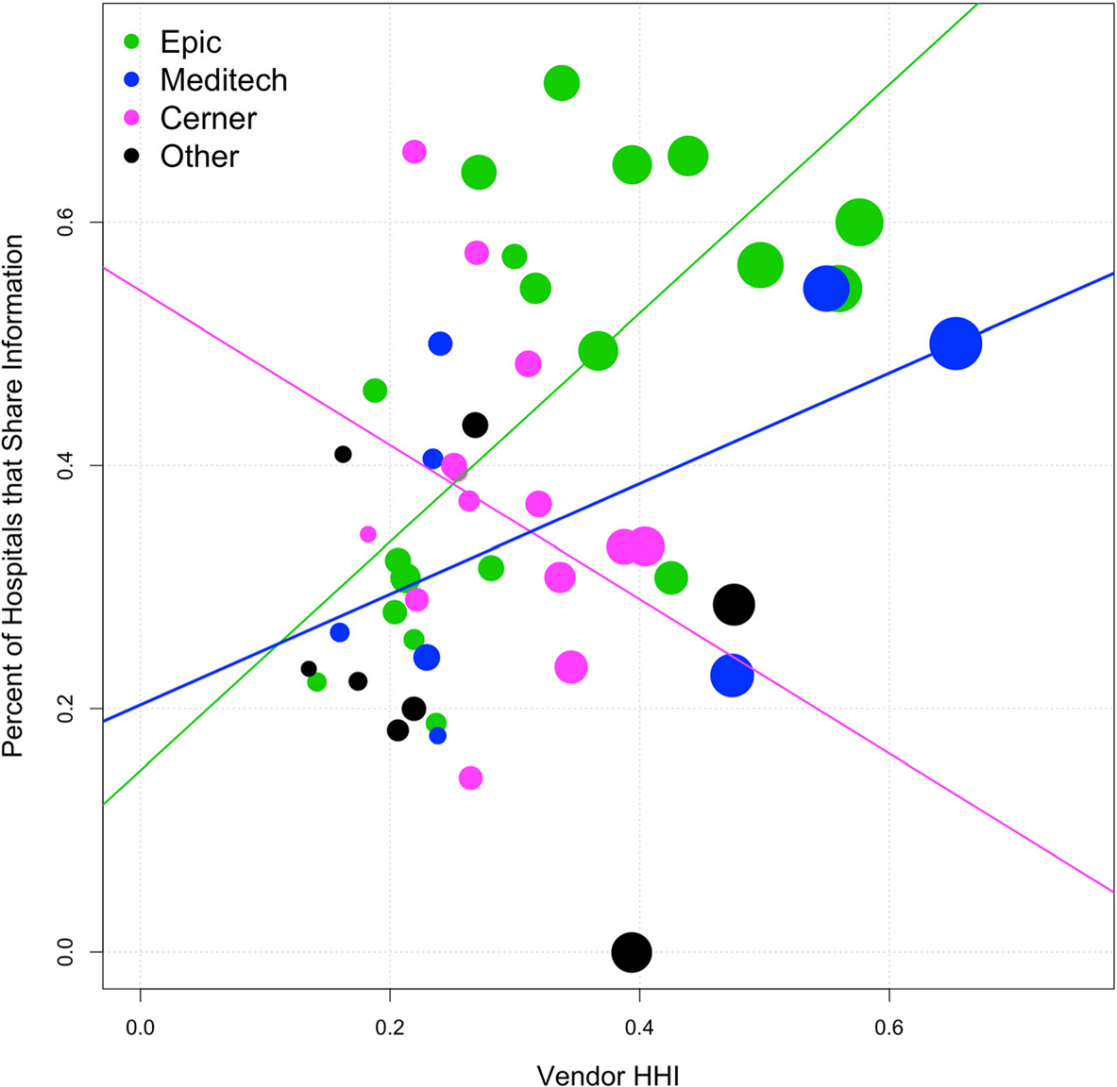
State Sharing versus Vendor HHI with dominant EHR vendor

Our second analysis examines at the market dynamics of EHR vendors at the state level and state policies to incentivize information exchange. The percentage of sharing varies widely across states, with Florida, Illinois, Missouri, New Mexico, Oklahoma, Tennessee and Texas sharing significantly less than the global mean of 37% (see Additional file 1). The differences across states have been attributed to factors such as state-level privacy regulation and information security practices [17, 35]. Another possible explanation is the different strategic approaches for information exchange prompted by the incentives received through the State Health Information Exchange Cooperative Agreement Program. We find no support for different strategies accounting for different levels of sharing. However, this study does find that these differences could also be explained in part by differences in the market concentration of EHR Vendors across states. Figure 3 shows the relationship between Vendor HHI and the total sharing within a state with different colors for the dominant vendor in each state. In this figure, the size of the point is proportional to percent market share of dominant vendor and trend lines indicate linear relationship between Vendor HHI and Percent of Hospitals that Share Information. We find that the three trend lines for each vendor mirror the relationship found at the hospital level. However, because of the smaller sample at the state level for each vendor this relationship is only statistically significant for states in which the dominant vendor is Epic.

Our results show that there is a relationship between EHR vendor concentration in a state and the percentage of hospitals that participate in the exchange of clinical care summaries within a state. Table 4, column 1 shows the result of the base bivariate linear regression model. Our independent variable of interest, Vendor HHI, is positively related to the percent of hospitals that participate in information exchange within a state. In our sample, as the market concentration of EHR Vendors in a state increases there is also an increase in the percent of hospitals that exchange clinical care summaries. This value remains significant as we include control variables such as the market concentration of hospitals within a system (column 2), the availability of Directed Exchange or Query Exchange within a state (column 3), and, the strategies used by the state as part of the State Health Information Exchange Cooperative Agreement Program (column 4).

**Table 4:** State level linear regression with dependent variable “Percentage of Hospitals in State that Share Clinical Care Summaries”

	Dependent variable:			
	Percentage that Share Clinical Summary			
	(1)	(2)	(3)	(4)
Vendor HHI	0.349^**^	0.657^***^	0.636^***^	0.639^***^
	(0.168)	(0.188)	(0.193)	(0.202)
System HHI		− 0.714^**^	− 0.900^**^	− 0.948^***^
		(0.242)	(0.292)	(0.366)
No. of Hospitals			−0.209	−0.347^*^
			(0.145)	(0.172)
QE Statewide			−0.045	
			(0.048)	
DE Statewide			−0.016	
			(0.065)	
Elevator				0.073
				(0.073)
Public Utility				0.042
				(0.083)
Capacity Builder				0.116
				(0.091)
Orchestrator				0.081
				(0.065)
Constant	0.280^***^	0.289^***^	0.410^***^	0.308^***^
	(0.061)	(0.058)	(0.099)	(0.099)
Observations	49	49	49	49
R^2^	0.08	0.23	0.27	0.30
Adjusted R^2^	0.06	0.20	0.19	0.19
Residual Std. Error	0.17 (df = 47)	0.15 (df = 46)	0.16 (df = 43)	0.16 (df = 41)
F Statistic	4.3^**^ (df = 1; 47)	6.9^***^ (df = 2; 46)	3.3^**^ (df = 5; 43)	2.6^**^ (df = 7; 41)

Note:^*^p < 0.1; ^**^p < 0.05; ^***^p < 0.01

In column 2 we see that including the System HHI variable has an effect on the marginal value of our key variable of interest. This result is consistent with the fact that hospitals within a health system are likely to use a unique vendor and that the initial Vendor HHI effect might be related to health system concentration. Nevertheless, even correcting for this possible omitted variable bias, the Vendor HHI remains positive and significant. The negative nature of the coefficient on System HHI is consistent with previous research [17] which showed that states with larger networks dominating the market have a lower percentage of hospitals that participate in information exchange outside their health system.

## Discussion

Through this analysis we have found empirical evidence that, among other factors, vendor market share and concentration are related to the likelihood of a hospital sharing clinical care summaries and the percentage of hospitals within a state that exchange such information. These factors remain important even when we take into account policies that incentivize information exchange such as the requirement for the use of standardized formats (CCR, CDA and CCD) and State level programs. While the capability to use a common format to send clinical care summaries is significant in increasing the likelihood of participating in the exchange of these documents, this ability is not enough to guarantee exchange outside a hospital’s network. In fact, 72% of hospitals that do not share clinical care summaries with other vendors are capable of using these common formats. Furthermore, almost 30% of hospitals that can use CCD and CDA continue to claim that they are not capable of exchanging clinical care summaries with hospitals using a different certified EHR vendor. This supports the notion that even when EHR systems are certified to comply with this MU requirement, exchange with outside vendors remains a challenge.

In this context, EHR vendor market share and concentration become relevant topics of analysis. Of the hospitals that exchange clinical care summaries with hospitals outside their system 23% assert that they cannot exchange with hospitals using a different EHR vendor (despite the fact that only 10% of hospital EHR systems don’t support CCD or CDA exchange standards), suggesting that exchange in this subgroup is happening directly between hospitals using the same EHR vendor. Although we cannot conclude from the available data if exchange for the rest of the sample is taking place through proprietary or standards-based methods, we can presume that there are benefits to exchanging using proprietary methods that are strengthened when the vendors are more concentrated. These benefits may include reduced technical difficulty and ease of access to specific interfaces, which might remain influential even if a hospital is technically able to exchange using standard formats.

When we control for each of the three largest EHR Vendors in the market we find relevant differences in the propensity for information exchange. We analyze the interactions with these EHR vendors in our sample and find that the positive relationship between HIE and market share is very strong for hospitals that use Epic. Hospitals using EHR vendor Epic are much more likely to exchange clinical care summaries than the rest of the hospitals in our sample. Conversely, hospitals that use Meditech and Cerner are less likely to exchange this type of information. By promoting proprietary sharing, larger players strengthen the network externality benefit of choosing an EHR from a large player. From these results we can infer that the availability of Epic’s Care Everywhere has important implications for hospitals looking to participate in information exchange. In fact, Epic becomes an interesting case study for the effects of having a proprietary network for health information exchange. Our analysis shows that Epic users might overcome some of the barriers for information exchange when other hospitals in the same region use Epic. However, when there are no users nearby that use this same EHR vendor the net benefits for exchange are diminished. This suggests that when removing the incentive of a geographically close Epic user for exchange, additional customization could act as a deterrent for developing further HIE capabilities [27, 28].

Due to the competitive nature of the EHR market, a larger player such as Epic could leverage its network size by facilitating intra-vendor sharing in an effort to enlist new users interested in sharing within its existing network. Smaller practices and hospitals interested in exchanging clinical care summaries with larger hospitals that use said EHR vendor would need to join the network. The decision to choose a specific EHR product involves a lock-in factor because of the sizeable costs of implementation. Not only does this make it unlikely that smaller hospitals could then change to a different vendor, it may involve additional unforeseen costs that could discourage them from implementing usable exchange capabilities [10, 36].

We have similar results at the state level. We find that higher Vendor HHI is positively correlated with the percentage of hospitals within the state that share information, even when controlling for different policies that incentivize or hinder information exchange. The different strategies applied through the State Health Information Exchange Cooperative Agreement Program (State HIE) do not show a significant effect on the percentage of hospitals that exchange clinical care summaries within a state. Hence, in states with highly concentrated markets measured by the Herfindahl-Hirschman Index (where one or two EHR vendors are used by the majority of the hospitals) there are more hospitals engaging in information exchange.

Part of the objective of the State HIE program was to fill HIE service gaps and build capacity for every eligible provider [5]. The fulfillment of this goal could be an important contribution toward overcoming some of the limitations of vendor facilitated exchange and the possible failures of closed proprietary networks. Unfortunately, our current research shows that none of the state level strategies seem to be successful in reducing this effect. In states where there are less concentrated markets, none of the different implementations were significant in incentivizing exchange. This might be a symptom of misaligned incentives, as there have been reports of current regulation undermining the role of community health information exchanges supported by State HIE by allowing EHR vendor mediated exchange that cuts out public exchanges [10].

As more hospitals transition to the second stage of meaningful use, data from recent years shows that similar challenges for HIE persist. While the percentage of hospitals that report that they have the capability to send clinical care summaries has increased, the percentage of hospitals that send them during transitions of care remains low. Data from Meaningful Use attestations between 2014 and 2016 shows that a median hospital sends clinical care summaries electronically for 33% of transitions, while the use of Epic as an EHR provider positively increases this probability [37]. Furthermore, qualitative work evidences that the number of EHR providers in the market, and the need for different interfaces to exchange clinical information between them, is still reported as an important barrier for HIE [10]. A recent survey of third party HIE organizations supports the issues of vendor information blocking, with half of those surveyed reporting that they had experienced information blocking by an EHR vendor [38]. Finally, vendor choice remains an important determinant in the successful implementation of MU objectives [11, 39].

## Limitations

There are some important limitations to our results. First, data from the AHA IT Supplement is self-reported and has limited representativeness with a self-selected sample of 61% of the population. While this database has been validated for reliability against other sources, it does show some bias toward over reporting [40]. It also includes some responses that are inconsistent and were removed from the dataset. Both of these issues would likely result in an overestimate of our measure of interoperability. Additionally, although we aimed to include most variables relevant to our analysis, there are other factors related to health information exchange that we were not able to quantify for this analysis. For example, we are not able to measure different security or privacy policies for different vendors that might facilitate or deter information exchange. Similarly, although research has found a relationship between state privacy policies and state information exchange practices, we were not able to include a measurement of privacy legislation in this study. It is possible that including indicators for state privacy regulation would have accounted for lower levels of information exchange. Third, we were only able to infer that EHR vendors in our analysis use proprietary methods for exchange because we do not have detailed information on the methods of information exchange for each hospital. Therefore, if a large percentage of hospitals are exchanging information through non-vendor mediated methods or regional health information exchanges, it is possible that some vendors offer an advantage for this type of sharing. Finally, all of our results show association and not causality because of the nature of the sample and the method.

## Conclusions

Identifying the barriers for information exchange is a necessary step to achieve the goals of the HITECH Act in creating a more efficient and effective healthcare system. Our research finds a relationship between the existence of dominant EHR networks and the exchange of clinical care summaries, which has important policy implications as the meaningful use program continues to transition to future stages. In fact, there is some evidence that information blocking could be partly the result of vague policies that undermine public exchanges.

Even though the current certification process for EHR products requires the use of a common language, there are several gaps that permit variability in its implementation. These gaps allow EHR vendors to implement information exchange capabilities in different ways. A clear example is the implementation of Care Everywhere, which has been successful in increasing sharing among Epic users. Nevertheless, the existence of isolated networks means that many hospitals are left out. In the case of Epic, this affects smaller and rural hospitals disproportionally (only 21% of hospitals that use EHR vendor Epic are rural which is significantly less than the sample mean).

In order to avoid proprietary exchange networks that foreclose some hospitals, it is important for the current regulation attempt to be more inclusive of hospitals that do not use large vendors and are therefore unable to use proprietary methods for exchange. Incentives could be tied to open exchange using previously defined standards rather than metrics that just measure if HIE occurs. For state level incentives, it might be necessary that state programs identify hospitals that are being left out of the exchange networks and offer technical and financial support. In our analysis at the state level we find no significant relationship between the percentage of hospitals that participate in health information exchange and the policies implemented through the State Health Information Exchange Cooperative Agreement Program. Our research suggest that future state level policies should take into account the different market conditions of EHR vendors in order to accommodate hospitals that may be left out of large proprietary networks.

Finally, although our findings suggest the importance of a network where information is exchanged only among hospitals that use a specific EHR vendor within a region and a state, further research is necessary to validate this relationship. Current information collection efforts only ask if information exchange occurs. More work needs to be done to determine the methods of exchange, including interviews with hospital staff that might give us some insight on if and why proprietary methods of exchange are being used.

## Additional file

Additional file 1: Additional Descriptive Statistics. Table A contains descriptive statistics of the data used and Table B contains state level data for information exchange. (DOCX 28 kb)

## Abbreviations

AHA: American Hospital Association
CCD: Continuity of Care Document
CCR: Continuity of Care Record
CMS: Centers for Medicare and Medicaid Services
EHR: Electronic Health Record
HHI: Herfindahl-Hirschman Index
HIMSS: Healthcare Information and Management Systems Society
HITECH: Health Information Technology for Economic and Clinical Health
HRR: Hospital Referral Region
IDS: Integrated Delivery System
IE: Information Exchange
MU: Meaningful Use
ONC: Office of the National Coordinator for Health Information Technology
RHIO: Regional Health Information Organizations
VMS: Vendor Market Share
