# Supplementary material for: Vendor of choice and the effectiveness of policies to promote health information exchange
Source: BMC Health Serv Res. 2018 Jun 4;18:405. doi: 10.1186/s12913-018-3230-7 (PMC5987601; doi:10.1186/s12913-018-3230-7)
Supplement: Supplementary file 1 — Additional Descriptive Statistics. Table A contains descriptive statistics of the data used and Table B contains state level data for information exchange. (DOCX 28 kb) [file 12913_2018_3230_MOESM1_ESM.docx]

Additional Descriptive Statistics

**Table S1**

Characteristics of Sample Hospitals for Logistic Regression Model

|  |  | *Sample Hospitals* | *Percentage*  *(N=1871)* |
| --- | --- | --- | --- |
| Hospital Size | Small (<100 beds) | 870 | 47% |
|  | Medium (100-399 beds) | 754 | 40% |
|  | Large (>=400 beds) | 247 | 13% |
|  |  |  |  |
| Ownership | Non-Profit | 1321 | 71% |
|  | For-Profit | 155 | 8% |
|  | Public | 395 | 21% |
|  |  |  |  |
| Affiliated to an IDS | Yes | 1135 | 60% |
|  | No | 672 | 36% |
|  | No Information | 64 | 3% |
|  |  |  |  |
| Capable of Using CCR, CDA or CCD | Yes | 1486 | 79% |
|  | No | 183 | 10% |
|  | Do Not Know | 202 | 11% |
|  |  |  |  |

**Table S2**

Percent of Hospitals that Share Clinical Care Summaries with Other Hospitals Outside their Health System

(Null hypothesis is that the difference in percent share is zero)

| State | % Share Clinical Care Summary | n(N) | Reject Null Hypothesis  p value* |
| --- | --- | --- | --- |
| Alaska | 33% | 2(6) |  |
| Alabama | 48% | 15(31) |  |
| Arkansas | 26% | 9(35) |  |
| Arizona | 33% | 11(33) |  |
| California | 32% | 47(148) |  |
| Colorado | 57% | 24(42) | p<0.05 |
| Connecticut | 31% | 4(13) |  |
| District Of Columbia | 0% | 0(4) |  |
| Delaware | 67% | 2(3) |  |
| Florida | 23% | 19(82) | p<0.05 |
| Georgia | 40% | 17(43) |  |
| Hawaii | 55% | 6(11) |  |
| Iowa | 29% | 20(70) |  |
| Idaho | 47% | 9(19) |  |
| Illinois | 19% | 22(118) | p<0.05 |
| Indiana | 46% | 24(52) |  |
| Kansas | 38% | 30(78) |  |
| Kentucky | 31% | 18(59) |  |
| Louisiana | 29% | 11(38) |  |
| Massachusetts | 24% | 8(33) |  |
| Maryland | 36% | 10(28) |  |
| Maine | 37% | 7(19) |  |
| Michigan | 31% | 20(65) |  |
| Minnesota | 56% | 57(102) | p<0.05 |
| Missouri | 21% | 24(112) | p<0.05 |
| Mississippi | 22% | 6(27) |  |
| Montana | 23% | 5(22) |  |
| North Carolina | 56% | 27(48) | p<0.05 |
| North Dakota | 54% | 7(13) |  |
| Nebraska | 43% | 13(30) |  |
| New Hampshire | 55% | 6(11) |  |
| New Jersey | 64% | 27(42) | p<0.05 |
| New Mexico | 14% | 3(21) | p<0.05 |
| Nevada | 18% | 2(11) |  |
| New York | 40% | 36(89) |  |
| Ohio | 63% | 55(87) | p<0.05 |
| Oklahoma | 19% | 9(48) | p<0.05 |
| Oregon | 71% | 15(21) | p<0.05 |
| Pennsylvania | 34% | 35(103) |  |
| Rhode Island | 0% | 0(7) | p<0.05 |
| South Carolina | 38% | 6(16) |  |
| South Dakota | 52% | 12(23) |  |
| Tennessee | 23% | 10(43) | p<0.1 |
| Texas | 27% | 53(199) | p<0.05 |
| Utah | 29% | 6(21) |  |
| Virginia | 64% | 25(39) | p<0.05 |
| Vermont | 50% | 3(6) |  |
| Washington | 65% | 22(34) | p<0.05 |
| Wisconsin | 50% | 47(94) | p<0.05 |
| West Virginia | 20% | 5(25) | p<0.1 |
| Wyoming | 31% | 4(13) |  |
| \| Note: \| \| --- \|   n = Number of hospitals that share clinical care summaries with hospitals outside their health system  N = Total number of hospitals per state in the database that responded to the variable of analysis  *Percentage share of other states is not significantly different than the total mean | | | |
